# microRNAs calibrate T cell responses by regulating mTOR

**DOI:** 10.18632/oncotarget.6107

**Published:** 2015-10-13

**Authors:** Matthias Merkenschlager, Antoine Marcais

**Affiliations:** CIRI, International Center for Infectiology Research, Université de Lyon, Inserm, CNRS, Ecole Normale Supérieure, Lyon, France

**Keywords:** microRNA, T cell activation, anergy, interleukin-2, mTOR

microRNAs are a class of small, non-coding RNAs that regulate gene expression at the post-transcriptional level. By blocking the stability and translation of protein-coding mRNAs, microRNAs impact most biological systems in health and disease, including the development and the function of the immune system [1]. A recent study by Marcais et al. has added new insights how microRNAs guide critical decisions by T lymphocytes [2].

T cells receive constant inputs from their surroundings - the challenge is that T cells must tolerate our own healthy cells and harmless commensals, yet mount vigorous immune responses against potentially harmful pathogens, transformed cells and other threats. If this discrimination fails, T cells may mistakenly attack healthy cells and cause autoimmunity, or they may fail to protect us from harm. A tried and tested strategy is that T cells tolerate signals that exclusively engage their T cell receptor (TCR), but go after signals that simultaneously engage the TCR and a set of co-stimulatory receptors that indicate the presence of tissue damage or inflammation [3]. In isolation, TCR signals trigger a state of unresponsiveness called anergy, whereas combined inputs through the TCR and co-stimulatory receptors trigger T cell activation, proliferation and the acquisition of effector functions [3]. The cytokine interleukin 2 (IL-2) is critical in this context: full T cell activation is required for T cells to produce IL-2. In turn, IL-2 promotes T cell activation and can override the induction of anergy [3].

To probe the role of microRNAs in T cell activation, Marcais et al. [2] generated CD4 T cells that were genetically deficient of the RNase III enzyme Dicer, which is required for the biogenesis of most microRNAs. Interestingly, these microRNA-deficient T cells became fully activated in response to isolated TCR signals, which would have rendered normal T cells anergic [2]. Analysis of the underlying mechanisms showed that the resistance of microRNA-deficient T cells to anergy was due to their ability to make and secrete IL-2 in the absence of co-stimulatory signals. As a result, microRNA-deficient T cells could even override anergy induction in normal bystander T cells through the paracrine action of IL-2 [2].

**Figure 1:**
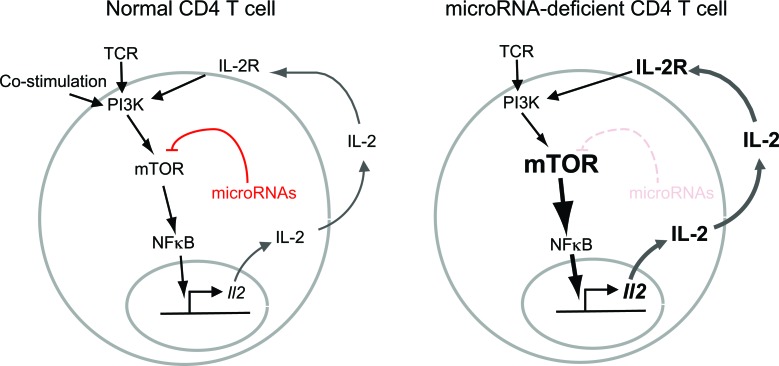
In wild type CD4 T cells, IL-2 secretion requires the coincidence of 2 signals through the TCR and through co-stimulatory receptors (left) In microRNA-deficient T cells, TCR signals are amplified by increased expression of Mtor components and increased mTOR activation (right). Through a series of intermediate steps that may involve NF-kappaB and other factors, this mechanism leads to IL-2 secretion in the absence of co-stimulatory signals and to autocrine and paracrine T cell activation.

Detailed analysis of the signaling machinery in microRNA-deficient T cells revealed several lesions, including the abnormally strong and prolonged activation of mTORC1 and mTORC2 (‘mTOR’ stands for ‘mechanistic target of rapamycin’ and ‘C’ stands for ‘complex’). mTOR complexes integrate signaling pathways to regulate a broad range of cellular functions. A number of mTOR complex components were already known targets of microRNA regulation in normal [4] and in cancer cells [5], and the authors demonstrated that the expression of Mtor and Rictor was regulated by Let-7, Mir-16 and most likely other microRNAs in T cells [2]. In the absence of microRNAs, Mtor and Rictor were overexpressed, but was this overexpression responsible for uncontrolled IL-2 production by micro-RNA-deficient T cells? To address this question, Marcais et al. [2] used genetics to introduce one defective *Mtor* allele and one defective *Rictor* allele into Dicer-deficient T cells. As a result, Mtor and Rictor proteins were derived from a single *Mtor* and a single *Rictor* allele. This approach compensated for the lack of post-transcriptional control, and restored near-normal levels of Mtor and Rictor proteins to microRNA-deficient T cells. Critically, reversing the overexpression of Mtor and Rictor also reduced the ability of microRNA-deficient T cells to produce IL-2 in the absence of co-stimulation [2]. This work supports the idea that microRNAs help T cells decide between activation and anergy, at least in part by controlling the precise expression of cellular signaling components (Figure 1).

In this context it is of note that recent data suggest an additional role for microRNAs in developing thymocytes, the precursors of mature T cells [6]. Here, microRNAs can make cellular responses more predictable by reducing cell-to-cell variability. Whether or not this principle applies in mature T cells has not yet been examined. Nevertheless, given that the inappropriate production of IL-2 can override anergy in bystander cells, it is interesting to reflect on the possibility that ‘outlier’ cells might destabilise immunological responses by inappropriately IL-2 production. However, sustained T cell expansion may require a critical number of activated T cells at the outset [7]. Hence, in addition to microRNAs, the immune system is ‘buffered’ by additional mechanisms that confer stability and ensure that important decisions are made based on consensus.
